# Association between overactive bladder and pelvic organ mobility as evaluated by dynamic magnetic resonance imaging

**DOI:** 10.1038/s41598-021-93143-6

**Published:** 2021-07-02

**Authors:** Kurenai Kinno, Noritoshi Sekido, Yasuharu Takeuchi, Yoshitomo Sawada, Shoutarou Watanabe, Yasukuni Yoshimura

**Affiliations:** 1 Department of Urology, Toho University Graduate School of Medicine, 5-21-16 Omorinishi, Ota City, Tokyo, 143-8540 Japan; 2 Department of Urology, Toho University Ohashi Medical Center, 2-22-36 Ohashi, Meguro City, Tokyo, 153-8515 Japan; 3 Department of Urology, Yotsuya Medical Cube, 7-7 Nibancho, Chiyoda City, Tokyo, 102-0084 Japan; 4 Female Pelvic Health Center, Showa University Northern Yokohama Hospital, 35-1 Chigasaki-chyuou, Tsuzuki Ward, Yokohama, Kanagawa 224-8503 Japan

**Keywords:** Urology, Bladder, Urological manifestations, Urogenital diseases

## Abstract

Overactive bladder (OAB) is a prevalent condition, which negatively impacts patients’ quality of life. Pelvic organ prolapse (POP), also prevalent in women, has been recognized as an important etiology of female OAB, although the pathophysiological mechanisms remain controversial. In this study, we reviewed findings of dynamic magnetic resonance imaging (dMRI) in 118 patients with POP and investigated the association between dMRI findings, including positions and mobilities of pelvic organs as well as parameters of pelvic organ support and bladder outlet obstruction (urethral kinking), and OAB in order to elucidate the pathophysiology of OAB in patients with POP. Our results showed that compared with non-OAB patients, OAB patients had a significantly higher body mass index, more severe pelvic floor muscle impairment, and more profound supportive defects in the uterine cervix (apical compartment). On the other hand, dMRI parameters showed hardly any significant difference between patients with mild and moderate to severe OAB. These findings may imply that levator ani impairment and defective supports of the apical compartment could be associated with the presence of OAB and that the severity of OAB could be affected by factors other than those related to pelvic organ mobility and support or urethral kinking.

## Introduction

Overactive bladder (OAB) is a prevalent condition that deteriorates the quality of life (QOL) of patients^1^. OAB has diverse etiologies, which include supra-sacral neurological diseases, metabolic syndrome, autonomic dysfunctions, and bladder outlet obstruction (BOO)^1^. In addition, a decreased level of estrogen, bladder outlet incompetence, and especially, pelvic organ prolapse (POP) are associated with the pathophysiology of female OAB^2^. The prevalence of OAB in women with and without POP is 22.5–52.0% and 2.9–25%, respectively, in community-based studies, which indicates that the relative risk is 2.1 to 5.8. On the other hand, the prevalence is 16–88% and 14–64%, respectively, in hospital-based studies, which indicates that the relative risk is 1.1 to 3.4^2^. Although the pathophysiology of OAB in women with POP is unclear, POP causes OAB through various mechanisms, such as BOO due to urethral kinking, premature activation of stretch receptors due to bladder wall distension, and urinary flow into the urethra due to urethral incompetence, all of which are associated with afferent excitation and detrusor overactivity (DO)^2,3^.

POP is also a prevalent disease and negatively affects QOL of patients. Because POP repair ameliorates OAB symptoms in up to 60% to 80% of patients^4,5^, POP is certainly associated with OAB. However, the association between the severity as well as the compartment of POP and OAB remains to be determined^6,7^. An Overactive Bladder Questionnaire of patients with POP Stage IV showed significantly worse scores than in those with POP stage II^4^, while OAB is more severe in patients with POP stage II rather than in those with POP stage III or IV^5^. In addition, some researchers suggested that OAB symptoms were not associated with vaginal descent or POP stage^6,8^. Regarding the association between the compartment of POP and OAB, patients with posterior/apical prolapse tended to have more moderate to severe OAB complaints than those with anterior/apical prolapse^5^. Takazawa et al.^9^ reported that a tension-free vaginal mesh procedure using a minimal mesh that mainly supports the apical compartment resolved OAB symptoms in 78.4% of patients with OAB. These findings indicate that it remains to be determined which compartment should be repaired in order to sufficiently improve OAB. To this end, investigating the association between the condition of pelvic organs as well as their supporting tissues and OAB by imaging studies may shed light on the pathophysiology of OAB in patients with POP, which may guide the selection of appropriate treatment.

Recently, it is growing evidence that without radiation exposure, dynamic magnetic resonance imaging (dMRI) clearly outlines the compartment and degree of POP during abdominal straining, pelvic organ mobility (POM), and impairment in levator ani as well as associated supportive tissue^10–19^. Alt et al. proposed that POM measured by dMRI be used as one of the treatment outcomes after pelvic floor reconstructive surgery^11,19^, and showed that POM reflected QOL associated with bowel dysfunctions^19^. However, the relationships between pelvic organ positions as well as POMs on dMRI and OAB have not been reported. In this study, to elucidate the pathophysiology of OAB in patients with POP, we investigated the association between parameters including pelvic organ positions and POMs derived from dMRI findings and the presence of OAB as well as its severity by the use of an OAB symptom score, OABSS^20^.

## Results

The mean age of the patients was 60.25 years old [95% confidence interval (CI) 58.27, 62.24], and the mean body mass index (BMI) was 22.91 kg/m^2^ (95% CI 22.36, 23.46). Twenty-nine patients (24.6%) complained of pelvic pressure, while 89 patients (75.4%) complained of vaginal bulging. Physical examination at the outpatient clinic revealed that 4, 32, 80, and 2 patients had stage I, II, III, and IV POP, respectively. Mean and median total OABSS were 3.13 (95% CI 2.59, 3.66) and 2.0 (interquartile range: 1.0, 4.25), respectively.

### Findings by presence or absence of OAB

Thirty-five (29.7%) patients had OAB, and patient characteristics by the presence or absence of OAB are shown in Table 1. Compared with non-OAB patients, OAB patients showed significantly higher BMI and a lower proportion of patients who smoke.

**Table 1:** Characteristics of patients with or without overactive bladder (OAB).

	Non-OAB		OAB		p
	n = 83		n = 35		
	Mean	95% CI	Mean	95% CI	
Age, years	59.10	56.73, 61.46	63.00	59.34, 66.66	0.0750
BMI, kg/m^2^	22.34	21.68, 22.99	24.24	23.32, 25.16	0.0004*
Parity	1.96	1.78, 2.15	2.26	1.99, 2.53	0.0580*
Smoking, n	14		0		0.0097
**Chief complaints, n**					
Pelvic pressure	21		8		0.7782
Vaginal bulging	62		27		
**Co-morbidities, n**					
Diabetes	1		2		0.2095
Hypertension	18		14		0.0682
Hyperlipidemia	12		8		0.2898
**POP-Q stage, n**					
I/II/III/IV	4/24/54 /1		0/8/26/1		0.4359
OABSS total score	1.60	1.32, 1.89	6.74	5.88, 7.61	< 0.0001
SCIPP line, mm	114.33	112.56, 116.11	114.93	112.19, 117.67	0.7144

The p-value for numerical variables was calculated by Student’s t-test except the p-values with a superscript (*), for which the Wilcoxon rank sum test was used.

BMI, body mass index; CI, confidence interval; OABSS, overactive bladder symptom score; POP-Q, pelvic organ prolapse quantification system; SCIPP, sacrococcygeal inferior pubic point.

**Figure 1:**
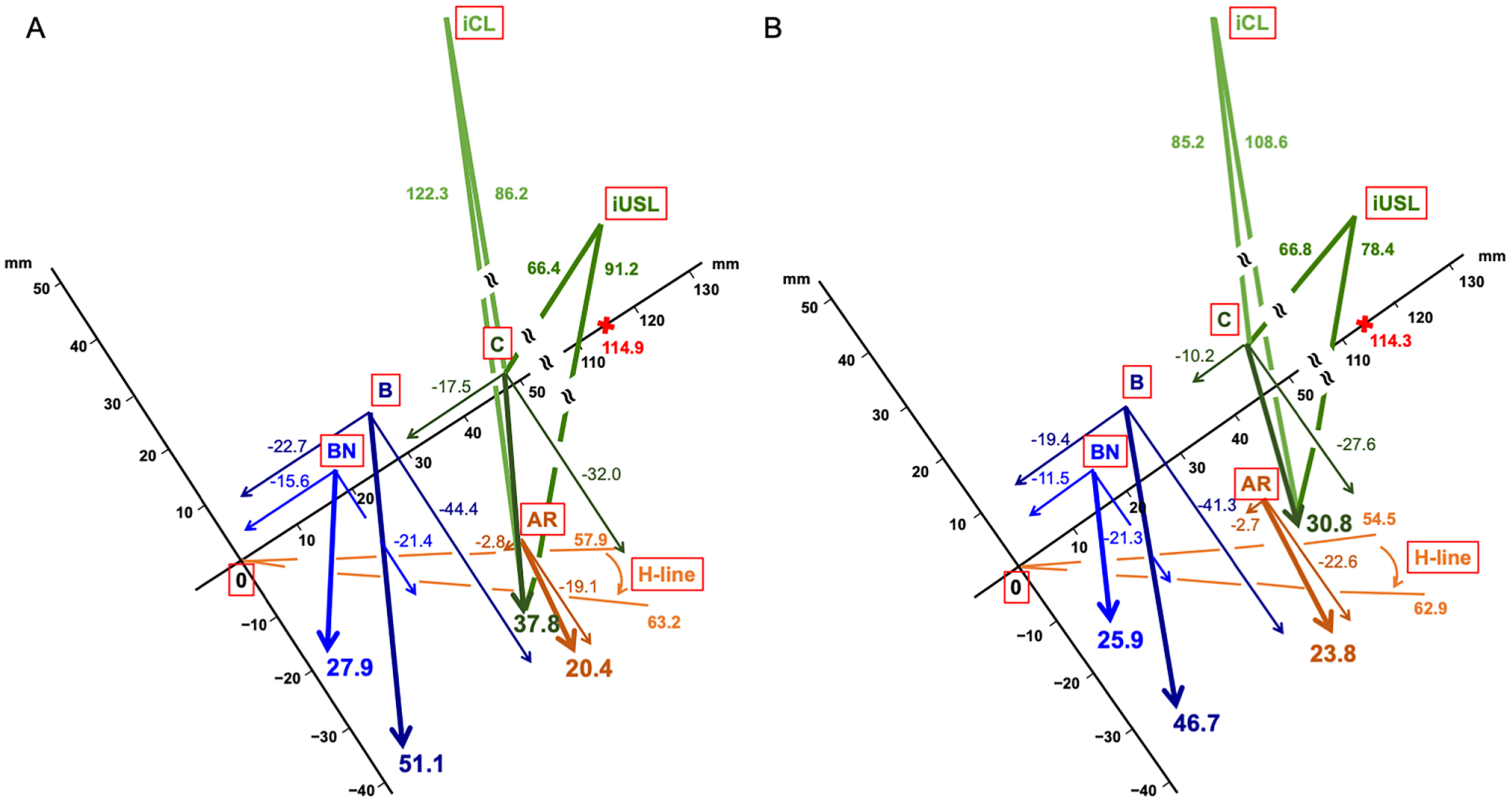
Graphic presentation of coordinate positions of pelvic organs at rest and during straining as well as mean values (mm) of pelvic organ mobilities during straining in patients with overactive bladder (OAB) (**A**) and without OAB (**B**). The sacrococcygeal inferior pubic point line (X-axis) and Y-axis are tilted counterclockwise based on the mean angle in relation to the bottom line on dynamic MRI images; non-OAB, 35.7° [95% confidence interval (CI) 31.3°, 37.0°]; OAB, 33.7° (95% CI 31.3°, 36.1°). To avoid further cluttering this figure, the hiatal descent and anterior vaginal wall length were not drawn on the figure. 0, inferior margin of the symphysis pubis; AR, anorectal angle; B, most dependent position of the bladder; BN, bladder neck; C, uterine cervix; H-line, length of the urogenital hiatus; iCL, imaginary line of the cardinal ligament; iUSL, imaginary line of the uterosacral ligament; X, sacrococcygeal joint.

**Figure 2:**
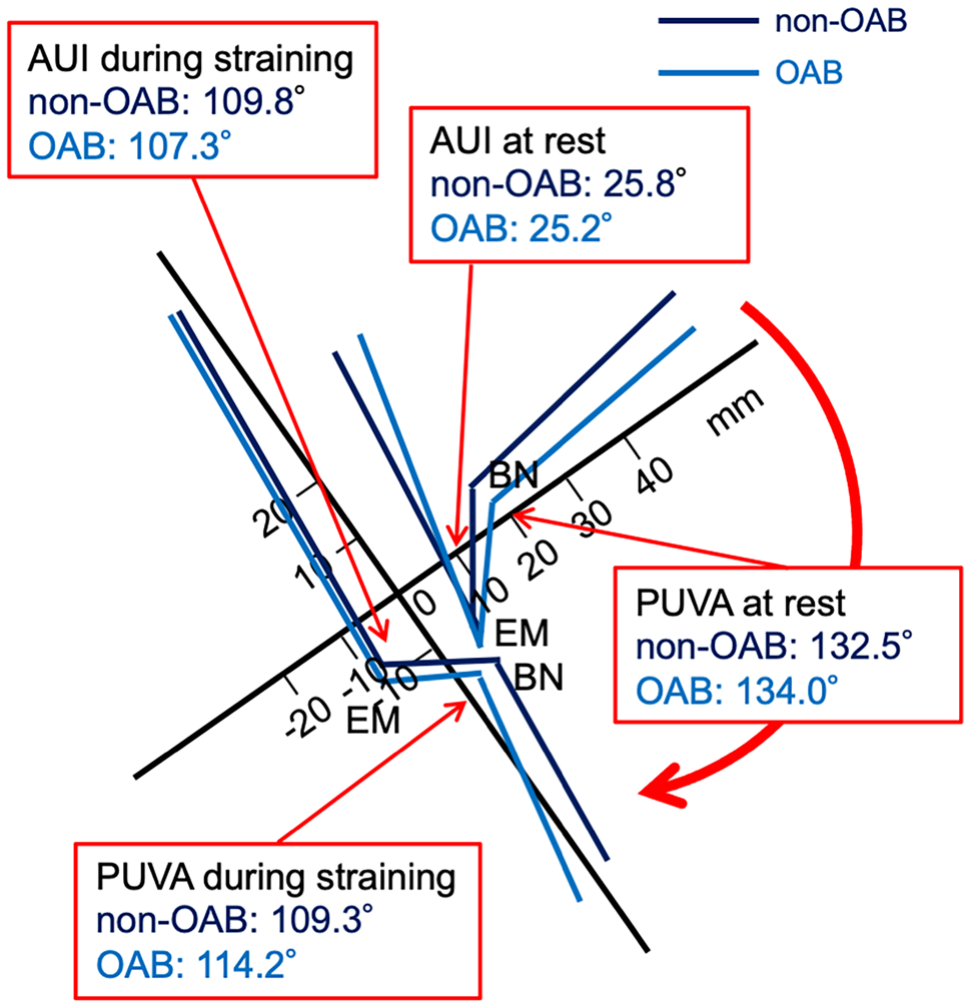
Graphic presentation of mean values (degree) associated with bladder outlet obstruction at rest and during straining in patients with or without overactive bladder (OAB). The sacrococcygeal inferior pubic point line (X-axis) and Y-axis are tilted counterclockwise based on the mean angle in relation to the bottom line on dynamic MRI images; all patients, 35.1° [95% confidence interval (CI) 33.9°, 36.3°]. 0, inferior margin of the symphysis pubis; AUI, angle of urethral inclination BN, bladder neck; EM, external urethral meatus; PUVA, posterior urethrovesical angle.

The findings of coordinate positions and POMs of pelvic organs as well as parameters of pelvic organ support and urethral kinking are shown in Table 2 and Figs. 1 and 2. In terms of coordinate points and POMs, OAB patients had significantly greater caudal uterine cervix (C) and anorectal angle (AR) at rest, and longer hiatal length at rest than non-OAB patients. During straining, C was located significantly more ventrally as well as obliquely downward in OAB patients than in non-OAB patients, which was reflected by the larger distance between the x coordinate positions (Cxx) as well as the larger diagonal distance (Cp) of C before and during straining. These movements of the apical compartment in OAB patients were accompanied by a significantly more extended imaginary uterosacral ligament (iUSL) as well as imaginary cardinal ligament (iCL) than in non-OAB patients. On the other hand, the degree of rotational descent of the urethra did not show a statistically significant difference between OAB patients and non-OAB patients. When the posterior urethrovesical angle (PUVA) was divided into groups below and above the upper limit of normal (115°)^21^, the proportion with OAB did not differ [29.9% (20/67) vs. 29.4% (15/51), respectively].

**Table 2:** Parameters on dynamic magnetic resonance imaging between patients with or without overactive bladder (OAB).

	Non-OAB		OAB		p
	n = 83		n = 35		
	Mean	95% CI	Mean	95% CI	
**BN, mm**					
At rest					
BNx	17.57	16.40, 18.74	18.96	17.09, 20.83	0.2038
BNy	5.61	3.76, 7.46	3.36	0.83, 5.89	0.1736
During straining					
BNx	6.09	4.20, 7.97	3.36	− 0.19, 6.91	0.1184*
BNy	− 15.70	− 17.45, − 13.95	− 17.99	− 21.02, − 14.96	0.0903*
Distance					
BNxx	− 11.48	− 13.71, − 9.25	− 15.60	− 19.55, − 11.65	0.0564
BNyy	− 21.31	− 23.02, − 19.60	− 21.35	− 23.95, − 18.74	0.9810
BNp	25.94	24.03, 27.86	27.90	24.33, 31.47	0.2988
**B, mm**					
At rest					
Bx	26.80	25.29, 28.31	28.37	25.82, 30.93	0.2718
By	10.25	8.12, 12.39	8.09	5.48, 10.70	0.2457
During straining					
Bx	6.98	4.43, 9.53	5.68	0.65, 10.72	0.6112
By	− 30.99	− 35.18, − 26.81	− 36.26	− 44.32, − 28.20	0.2052
Distance					
Bxx	− 19.44	− 22.46, − 16.41	− 22.69	− 28.60, − 16.78	0.2808
Byy	− 41.25	− 45.00, − 37.49	− 44.35	− 52.08, − 36.62	0.4170
Bp	46.67	42.37, 50.97	51.06	42.14, 59.97	0.3137*
**C, mm**					
At rest					
Cx	48.20	45.46, 50.94	48.42	43.71, 53.13	0.9324
Cy	7.84	5.75, 9.94	1.86	− 1.65, 5.38	0.0011*
During straining					
Cx	37.96	34.43, 41.49	30.88	24.40, 37.36	0.0410
Cy	− 19.71	− 22.68, − 16.74	− 30.13	− 37.56, − 22.69	0.0020
Distance					
Cxx	− 10.24	− 12.74, − 7.74	− 17.54	− 21.84, − 13.24	0.0021*
Cyy	− 27.56	− 30.19, − 24.93	− 31.99	− 38.05, − 25.92	0.1172
Cp	30.78	27.76, 33.80	37.75	31.12, 44.37	0.0291
**AR, mm**					
At rest					
ARx	35.97	34.69, 37.25	37.08	35.12, 39.04	0.2555*
ARy	− 13.19	− 15.37, − 11.02	− 19.86	− 23.03, − 16.70	0.0020*
During straining					
ARx	33.32	31.58, 35.07	34.25	31.28, 37.22	0.5760
ARy	− 35.79	− 38.07, − 33.51	− 38.97	− 43.57, − 34.38	0.1683
Distance					
ARxx	− 2.65	− 3.94, − 1.35	− 2.83	− 5.00, − 0.65	0.8597*
ARyy	− 22.60	− 25.19, − 20.01	− 19.11	− 22.42, − 15.80	0.0870*
ARp	23.78	21.32, 26.24	20.40	17.17, 23.64	0.1014*
**iUSL, mm**					
At rest	66.75	63.56, 69.94	66.44	61.83, 71.05	0.9140
During straining	78.44	74.05, 82.82	91.19	83.51, 98.86	0.0023*
iUSL ε	0.18	0.14, 0.23	0.38	0.29, 0.48	0.0001*
**iCL, mm**					
At rest	85.18	82.08, 88.28	86.18	81.18, 91.18	0.7294
During straining	108.59	104.04, 113.13	122.29	114.22, 130.35	0.0021
iCL ε	0.28	0.24, 0.32	0.43	0.35, 0.51	0.0007
**H− line, mm**					
At rest	54.45	52.86, 56.04	57.90	55.45, 60.36	0.0198
During straining	62.89	60.96, 64.82	63.22	60.11, 66.32	0.8000*
**Mʹ− line, mm**					
At rest	27.28	25.76, 28.80	29.69	27.49, 31.90	0.0809
During straining	39.24	37.18, 41.30	40.68	37.32, 44.04	0.4551
**AVWL, mm**					
At rest	37.96	35.41, 40.51	38.01	33.35, 42.68	0.9826
During straining	75.13	68.85, 81.41	75.33	64.13, 86.53	0.6522*
Change	37.17	30.82, 43.52	37.32	26.88, 47.75	0.7977*
**PUVA, degree**					
At rest	132.54	126.87, 138.20	134.03	124.80, 143.25	0.7785
During straining	109.26	101.19, 117.33	114.17	100.86, 127.49	0.5167
Change	− 23.28	− 32.42, − 14.13,	− 19.85	− 36.80, − 2.90,	0.7012
**AUI, degree**					
At rest	25.81	23.66, 27.96	25.23	21.83, 28.63	0.7589*
During straining	109.83	107.25, 112.41	107.34	103.20, 111.48	0.1203*
Change	84.02	81.06, 86.99	82.11	77.21, 87.02	0.4934

x and y indicate the x and y coordinates of each pelvic organ point (AR, B, BN, and C). xx and yy indicate the distance between coordinate positions of the pelvic organ points before and during straining in x and y directions, respectively. p indicates the distance derived from the Pythagorean theorem. Note that the effect of pelvic organ mobility (POM) on the X-axis is negative when moving in the ventral direction and that of POM on the Y-axis is negative when moving in the caudal direction. Also, note that all diagonal POMs are positive. The p-value for numerical variables was calculated by Student’s t-test except the p-values with a superscript (*), for which the Wilcoxon rank sum test was used.

AR, anorectal angle; AUI, angle of urethral inclination; AVWL, anterior vaginal wall length; B, most dependent position of the bladder; BN, bladder neck; C, uterine cervix; CI, confidence interval; H-line, length of the urogenital hiatus; iCL, imaginary line of the cardinal ligament; iUSL, imaginary line of the uterosacral ligament; M′-line, length of the hiatal descent; PUVA, posterior urethrovesical angle; ε, strain.

Of variables showing a statistically significant difference in Table 2, BMI, the y coordinate of AR (ARy) at rest, and strain on iUSL (iUSLε) were selected for multivariable logistic regression analysis. Odds ratios (ORs) of BMI, ARy at rest, and iUSLε for the presence of OAB were 1.13 (95% CI 0.97, 1.32), 0.95 (95% CI 0.90, 1.01), and 10.37 (95% CI 1.46, 73.45), respectively (see Model 1 of Supplementary Table S1 on line). When age was added to this model, it did not affect the results, which showed a statistical difference only in odds ratio (OR) of iUSLε [10.30 (95% CI 1.45, 73.44)] (see Model 2 of Supplementary Table S1 online).

### Findings by severity of OAB

Among OAB patients, the proportion of patients with hypertension tended to be higher in patients with moderate to severe OAB than in those with mild OAB (p = 0.0697, see Supplementary Table S2 online). In addition, patients with moderate to severe OAB tended to be older and have a higher BMI than those with mild OAB (p = 0.0949 and p = 0.0787, respectively, see Supplementary Table S2 on line). On the other hand, of the parameters on dMRI, only the x coordinate of the most dependent portion of bladder (Bx) at rest showed a significant difference, and it was located in a more ventral position in patients with moderate to severe OAB than in those with mild OAB, while the change of the angle of urethral inclination (AUI) tended to be smaller in patients with moderate to severe OAB than in those with mild OAB (p = 0.0582, Supplementary Table S3 on line).

## Discussion

To the best of our knowledge, no study has reported the association between parameters on dMRI and OABSS in patients with POP. In the present study, approximately 30% of the patients had OAB. We demonstrated that BMI, smoking status, levator ani impairment, and defective supports of the apical compartment were associated with the presence of OAB. On the other hand, all but one (Bx) of the parameters on dMRI showed no significant difference between patients with mild OAB and those with moderate to severe OAB.

Considering the findings of AR and the length of the urogenital hiatus (H-line), levator ani impairment could predispose patients to OAB. In contrast to “at rest”, the indexes on AR as well as the length of the H-line “during straining” did not show any statistical differences between OAB and non-OAB patients. This suggests that levator ani impairment in our patients was severe enough to cause OAB, so that any further caudal movement of AR as well as extension of the urogenital hiatal length no longer affected the presence or absence of OAB. The levator ani impairment potentially causes pelvic organ descent and vaginal wall protrusion below the line through the hiatus^22^. Consequently, a portion of the anterior vaginal wall is exposed to the pressure differential between high intra-abdominal pressure and low atmospheric pressure, which generates tension in the vaginal wall and acts as a downward force that drags the vaginal wall and apex downward with the force becoming stronger in proportion to the exposed vaginal wall length^14^. Therefore, levator ani impairment could have a profound adverse effect on the apical supports. On the other hand, defects in apical supports are associated with larger cystocele^23^. Chen et al.^23^ reported that an 80% impairment in apical support resulted in a 33% larger cystocele size. Both the uterosacral ligament (USL) and cardinal ligament (CL) play a critical role in apical supports^23,24^. When these structures are compromised, the resultant larger cystocele, that is, larger anterior vaginal wall protrusion, develops^23^. Moreover, Petros et al. hypothesized that debilitated suspensory function of pubourethral and uterosacral ligaments weakened the musculo-elastic stretching mechanism of the vagina which supported the bladder base stretch receptors^25^. The consequent lax vagina is not sufficient to prevent activation of the bladder base stretch receptors^25–27^. Eventually, stretching of the bladder wall might trigger the stretch receptors in the urothelium, which causes a prematurely activated micturition reflex, leading to OAB^2,25–28^. This implies that female OAB may be derived from a ligamentous–fascial disorder remote from the symptomatic organ^16^, which could explain why supports for an apical compartment, rather than the positions and POMs of the bladder neck (BN) or the most dependent position of the bladder (B), are significantly associated with the presence of OAB in the present study. In this respect, the present results may be considered to be in line with the previously reported posterior fornix syndrome attributed to defects in apical supports, which consists of urgency/frequency, nocturia, chronic pelvic pain, and abnormal emptying^5^. In fact, compared with controls, patients with POP showed an increase in the deep USL length from resting to straining of 15 ± 12 mm vs. 7 ± 4 mm, while CL was 30 ± 16 mm vs. 15 ± 9 mm^24^, which would be compatible with our findings on iUSL and iCL. The present study showed that compared with non-OAB patients, positions of C were significantly more ventral as well as caudal and POMs of C were significantly greater in OAB patients. Moreover, strain on iUSL and iCL (iUSLε and iCLε, respectively) was significantly larger in patients with OAB. Takazawa et al.^9^ showed that the transvaginal surgery with mainly apical support significantly improved OABSS total score from 4 to 2 points, suggesting that the importance of providing support for level I in improving OAB. Liedl et al.^29^ demonstrated that cardinal/uterosacral ligament repair using a Tissue Fixation System for symptomatic apical prolapse ≥ stage II cured urgency incontinence, frequency, and nocturia in 85%, 83%, and 68% of patients, respectively. Also, an Elevate technique, which uses mesh arms attached to the sacrospinous ligaments to recreate apical ligamentous support, significantly improved all OAB symptoms^5^. Taken together, the levator ani impairment and worse apical supports would be a fundamental factor that is associated with the presence of OAB in patients with POP.

In addition to the abnormally triggered micturition reflex by anterior vaginal prolapse^2^, neurodegeneration in the bladder wall seen in OAB should be considered based on the findings on iUSL and iCL. The USL is situated in close proximity to the hypogastric nerve (HN) and inferior hypogastric plexus (IHP)^30^, and a deeper portion of USL as well as the neural part of CL involves autonomic fibers from HN and IHP, and a part of IHP itself^30^. Therefore, it is reasonable that peripheral autonomic fibers travelling to and from the bladder are assumed to be subjected to cyclic strain in POP, which might cause partial denervation of the bladder wall. In fact, the density as well as the diameters of nerves in USL and CL in women with POP were significantly reduced compared with those without POP^2,31^. To the best of our knowledge, it remains to be determined whether cyclic strain of USL and CL in POP affects autonomic nervous fibers morphologically and functionally. A rapid recovery of the peripheral somatic nervous function is generally shown after 4% to 21% stretching of peripheral nerves^32^, although 5–25% stretching can cause nerve rupture^33^. Assuming that the autonomic nerve fibers are strained to the same degree as those ligaments, the findings on iUSLε and iCLε suggest that the strain on the nerve fibers would exceed these proposed ranges. Taken together, the extension of USL and CL might develop partial denervation of the bladder, which induces OAB.

As far as PUVA and AUI are concerned, BOO would not be strongly associated with the presence or absence and the severity of OAB. BOO is often regarded as an important mechanism for developing OAB in patients with POP^2^, although the exact mechanism of BOO in POP is not fully understood^34^. The mechanisms of BOO include the urethral kinking or urethral and/or BN compression by the prolapsed organs, which has been diverted from the urinary continence mechanism in POP^34,35^. A rapid upswing in urethral pressure in any segment along the entire urethral length, the downward movement of the bladder and posterior urethra against the immobile distal urethra, or an intact PUVA with significant BN descent during straining in patients with POP not only provide continence but also cause BOO^36–38^. However, there have been no reports to date that have clearly demonstrated the exact site of urethral kinking or urethral compression during the voiding phase^34^. In the present study, the exact site of urethral kinking could not be identified on dMRI because the entire urethral morphology was difficult to evaluate on the midline sagittal plane during straining. Moreover, the intact PUVA, which is supposed to be one of the indicators of the urethral kinking in POP^38^, was not associated with the presence of OAB. Therefore, mechanical BOO due to the urethral kinking or urethral and/or BN compression would not fully explain a mechanism of OAB in patients with POP. In this regard, we may have to pay attention to a role of the pelvic floor in opening and closing of BN as proposed by Petros et al.^39,40^, which illustrates that selective and specific directional contractions of the pelvic floor muscles stretch the vagina against intact pubourethral and USLs to facilitate opening and closure of the urethra and BN. Later, Bush et al.^41^ used a finite element model and demonstrated that backward/downward-acting pelvic floor muscles enabled normal micturition. Damage in the ligamentous structures could diminish the action of pelvic floor muscles, which impairs adequate opening of the urethra and BN, so-called functional BOO^26^. In fact, apical sling operations improved abnormal bladder emptying as well as OAB in patients with POP^42,43^. Taken together, the present dMRI findings suggest that the levator ani impairment and supportive defects in the apical compartment potentially cause functional BOO, which might be more likely to be involved in OAB in patients with POP, rather than mechanical BOO.

Although parameters evaluated on dMRI would be associated with OAB, none of the parameters except Bx at rest was associated with the severity of OAB. On the other hand, hypertension tended to be associated with it, and an association between the severity of OAB and BMI as well as age was also suggested, which would indicate that the severity of OAB would be more affected by systemic factors such as neuroendocrine, vascular, or inflammatory processes than by POP itself^44,45^. Caution should be exercised with regard to interpreting the results because the number of patients with OAB was small in this study. While levator ani impairment and defective apical supports could be associated with OAB, the severity of OAB would be more affected by systemic factors than by POP itself, which might partly explain the inconsistent results of relationship between OAB and POP in the literature^4,5^. Further studies are needed to determine the relationship between effects of systemic factors and parameters evaluated in dMRI on the occurrence and severity of OAB.

This study has several limitations. First, dMRI was performed in a supine, rather than a standing, position because our magnetic resonance imaging (MRI) equipment did not allow upright examination. Also, we did not measure intra-abdominal pressure during the Valsalva maneuver. However, dMRI was conducted by one specialized and experienced technician with a consistent protocol, and we included only patients who were able to reproduce POP ≥ stage II during straining. Second, we measured POMs with coordinates of selected landmarks at rest and during straining. Strictly speaking, the coordinates at rest would not correspond to the “normal positions” of the patients. However, we do not have data on the “normal positions” because we have not performed dMRI on nulliparous women without any symptoms, and a definition of the “normal positions” on dMRI of Asian women has not been established. Moreover, POMs should be adjusted to the patient’s height or pelvic bone geometry^13,46^. However, use of these corrected methods is not widespread yet. Third, we included patients with POP ≤ stage II at rest and POP ≥ stage II during straining. Therefore, we did not evaluate the association between mild (< stage II during straining) POP and OAB as well as between severe (≥ III at rest) POP and OAB. Fourth, we have not taken MRIs of patients after POP repair. Therefore, we did not investigate whether decreased POMs after POP repair correlated with the improvement of OAB.

In conclusion, considering the parameters derived from dMRI, levator ani impairment and defective supports of the apical compartment were associated with the presence of OAB, while almost all parameters derived from dMRI were not associated with the severity of OAB, which could be affected by systemic factors.

## Patients and methods

This retrospective study was conducted under the approval of the Clinical Research Ethics Committee at Yotsuya Medical Cube (ID: YMCIRB-20R010) and also approved by the Treatise Certificate Committee at Faculty of Medicine, Toho University (ID: 2020-157), by which the Declaration of Helsinki adopted by the World Medical Association, Ethical Guidelines for Medical and Health Research Involving Human Subjects established by the Ministry of Health, Labour and Welfare, and other relevant acts were followed accordingly. According to the Ethical Guidelines, because the present study is a retrospective observational study that did not utilize human biological specimens, it is not necessarily required to obtain written or verbal informed consent from individual patients, but it is necessary to disclose the contents of the study and provide an opportunity for opt-out, which was followed by the Clinical Research Ethics Committee at Yotsuya Medical Cube in approving the present study.

### Patients

Patients referred to Yotsuya Medical Cube for management of POP have routinely undergone dMRI to investigate the severity as well as the compartment of POP. Of 334 patients undergoing dMRI between August 2015 and July 2016, we included 148 patients who had POP ≤ stage II at rest and POP ≥ stage II during dMRI because POMs in patients with POP ≥ stage III both at rest and during straining paradoxically reveal small values. We also excluded patients taking OAB medication, with only rectocele and the following co-morbidities or previous histories: neurogenic lower urinary tract dysfunctions, anti-incontinence surgery, POP surgery, intrapelvic gynecologic, gastrointestinal and urologic surgeries, pelvic radiotherapy, and gynecological diseases, e.g., endometriosis, large uterine myoma, and large ovarian cysts. Of 148 patients, we excluded 30 patients due to unevaluable dMRI, which resulted from vigorous body motion during a forced Valsalva maneuver, so that we finally included 118 patients in this study.

### Protocols of MRI

We referred to the MRI protocol described in the study by Larson et al.^12^. The dMRI (1.5T, GE Signa Excite) was conducted by one experienced technician specializing in evaluation of urogynecological patients and was performed in the late afternoon because POP was more easily reproduced than in the morning or early afternoon. Prior to the MRI, the technician instructed patients on how to strain effectively and sufficiently during dMRI. Patients were instructed to drink enough fluid to be conscious of a normal desire to void at dMRI. First, axial as well as sagittal static T2 images were obtained to evaluate the integrity of pelvic floor musculature and connective tissue and the presence or absence of incidental lesions of pelvic organs. Then, dMRI on the midline sagittal plane was performed with consecutive acquisition of MRI images, at rest (2 to 3 images, repetition time [TR] range, 3.2; echo time [TE], minimum; 10 mm slice thickness, 0 mm gap; 1 number of excitations [NEX]) and during the maximal Valsalva maneuver for 10 to 15 s (10 to 15 images TR range, 3.2; TE, minimum; 10 mm slice thickness, 0 mm gap; 1 NEX), to generate cinematic images in the supine position with knees elevated on a high pillow. Dynamic sequences were repeated several times while subjectively monitoring the sufficiency of the Valsalva maneuver. At dMRI, incontinence pads were used to decrease fear of or embarrassment about leakage to allow patients to strain enough.

### Methods to evaluate POM (Fig. 3A,B)

**Figure 3:**
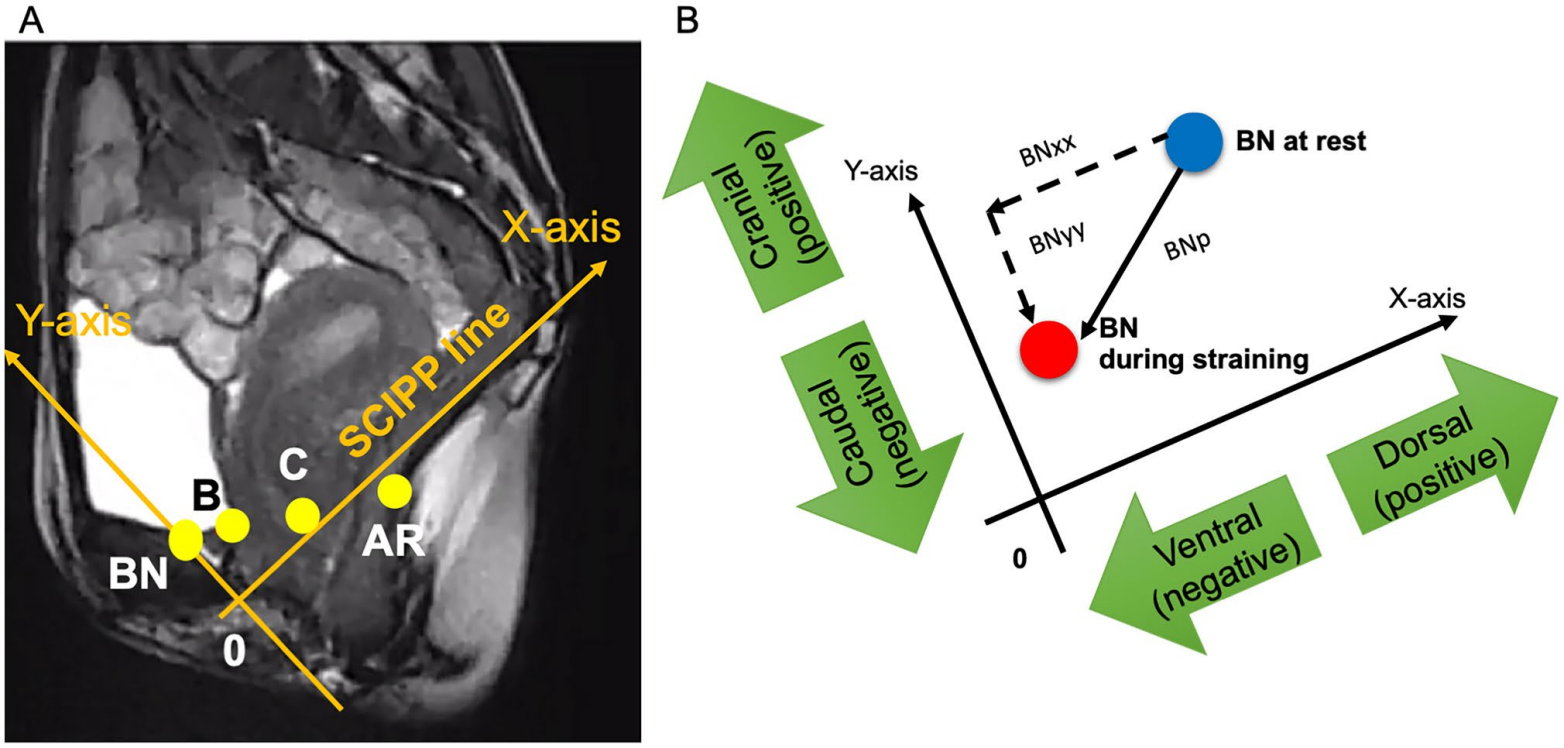
Methods to calculate positions and mobility of pelvic organs on dynamic magnetic resonance imaging. (**A**) Shows the selected coordinate positions of pelvic organs in the present study, while (**B**) shows how to calculate pelvic organ mobility, for example mobility of BN. As shown in (**B**), if BN moves ventrally in parallel with the X-axis, BNxx takes a negative value, while if BN moves dorsally, BNxx takes a positive value. Likewise, if BN moves caudally in parallel with the Y-axis, BNyy takes a negative value, while if BN moves cranially, BNyy takes a positive value. AR, anorectal angle; B, most dependent position of the bladder; BN, bladder neck; C, uterine cervix; xx, distance in x-direction; yy, distance in y-direction; X-axis corresponds to the sacrococcygeal inferior pubic point (SCIPP) line and Y-axis corresponds to a perpendicular line to X-axis at the origin (0, the inferior margin of the pubic symphysis).

Using the mid-sagittal dMRI images on the monitor, we measured representative points of the pelvic organs, that is, coordinate positions of the BN, B, C, and AR at rest and during intense straining^10,11,13,14^.

To measure each coordinate of these landmarks, we used a sacrococcygeal inferior pubic point (SCIPP) line connecting the inferior pubic point and the sacrococcygeal junction^15^ as an X-axis with the inferior margin of the pubic symphysis as an origin because its identification is more reproducible than the pubococcygeal line (PCL) on dMRI, and a line perpendicular to the SCIPP line at the origin as a Y-axis. The x and y coordinates of each representative point (e.g., BNx, and BNy, respectively) were measured before and during straining.

POM was evaluated by the distance between coordinate positions of the representative points before and during straining in x (e.g., BNxx = BNx during straining – BNx at rest), y directions (e.g., BNyy = BNy during straining – BNy at rest), and the distance derived from the Pythagorean theorem (e.g., BNp = [(BNxx)^2^ + (BNyy)^2^]^1/2^). Note that POM on the X-axis is negative when moving in the ventral direction and POM on the Y-axis is negative when moving in the caudal direction. Also note that all diagonal POMs are positive.

### Methods to evaluate parameters of pelvic organ support and bladder outlet obstruction (Fig. 4A,B)

**Figure 4:**
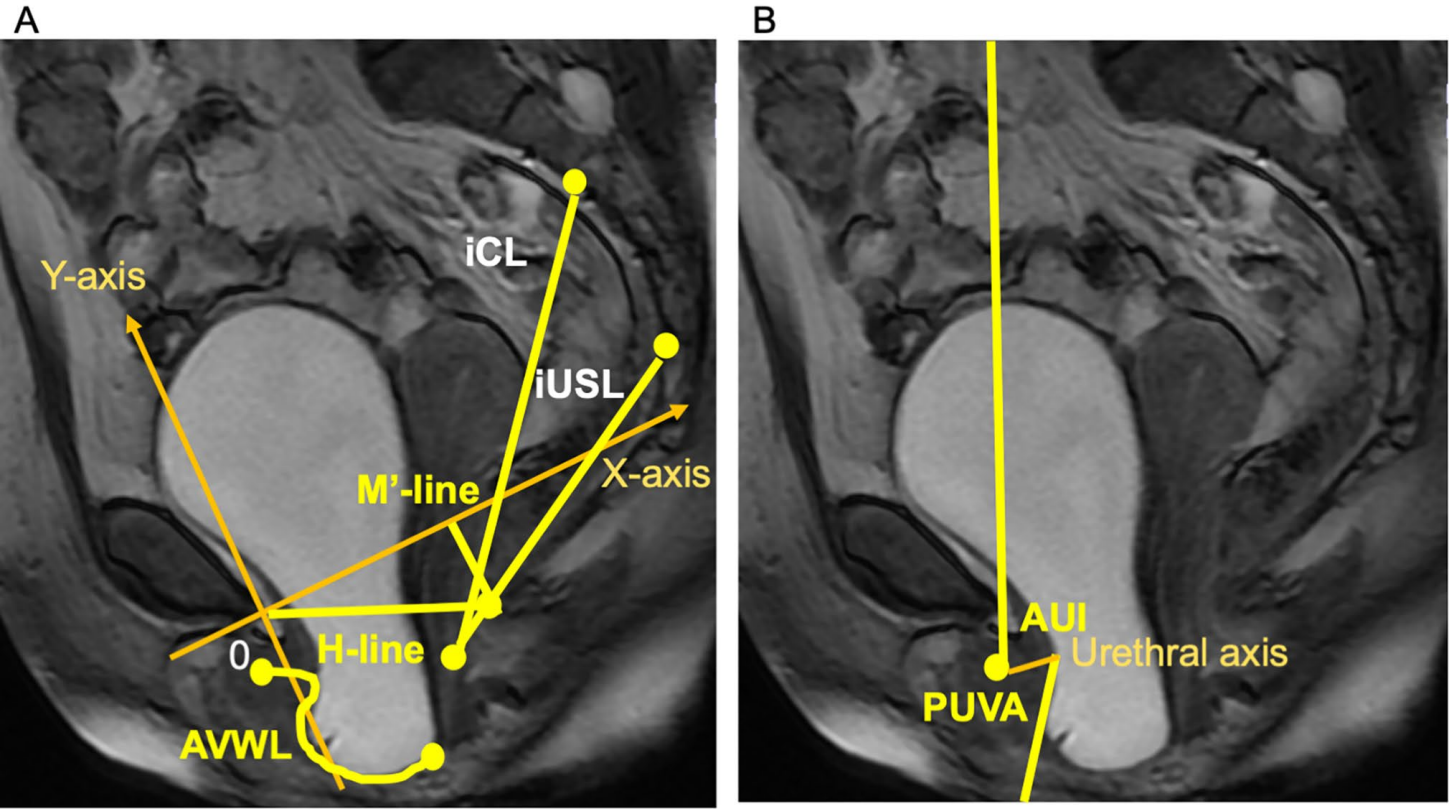
Methods to calculate parameters of pelvic organ support and bladder outlet obstruction on dynamic magnetic resonance imaging. (**A**) Shows measured parameters as follows: the anterior vaginal wall length (AVWL) is traced from the anterior vaginal fornix to the external urinary meatus; the length of the urogenital hiatus (H-line) is traced from origin to anorectal angle; the imaginary cardinal ligament (iCL) is traced from the anterior surface between the second and third sacral bones to the uterine cervix (C); the imaginary uterosacral ligament (iUSL) is traced from the anterior surface between the fourth and fifth sacral bones to C; the hiatal descent (M′-line) extends perpendicularly from the SCIPP line to the posterior end of the H-line. (**B**) Shows measured parameters for bladder outlet obstruction: the angle of urethral inclination (AUI) is the angle of the urethral axis in relation to the vertical plane; the posterior urethrovesical angle (PUVA) is the angle between the urethral axis and the posterior border of the bladder base or trigone.

We attempted to measure the length of the USL as well as the CL on dMRI. However, because it was difficult to precisely delineate the courses of these ligaments on the mid-sagittal images of dMRI, the distance from the anterior surface between the fourth and fifth sacral bones (S4/5) to C was measured as a surrogate of the USL as an iUSL^16^, while the distance from the anterior surface between the second and third sacral bones (S2/3) to C was measured as a surrogate of the CL as an iCL^17,47^. The lengths of those imaginary lines were measured at rest and during straining, from which we calculated the strain on them (e.g., iUSL ε = [the length of iUSL during straining—the length of iUSL at rest]/the length of iUSL at rest). We also measured the H-line and of the hiatal descent at rest and during straining^10^. Because we used the SCIPP line rather than PCL as a reference line, the hiatal descent (Mʹ-line) extended perpendicularly from the SCIPP line to the posterior end of the H-line. Moreover, the anterior vaginal wall length (AVWL) at rest and during straining, which was traced from the anterior vaginal fornix to the external urinary meatus^48^, was also measured. The morphology of the entire urethra could not be precisely delineated in dMRI due to its movement during straining. Therefore, using the following parameters, we evaluated urethral kinking as well as mobility: the PUVA, which is the angle between the urethral axis and the posterior border of the bladder base or trigone, and the AUI, which is the angle of the urethral axis in relation to the vertical plane^21^. From these variables, changes in AVWL, PUVA, and AUI were also calculated.

### Methods to evaluate OAB

The Japanese Clinical Guidelines for Overactive Bladder Syndrome strongly recommend the use of OABSS for diagnosis as well as evaluation of the severity and treatment outcomes of OAB^49^. In fact, several recent large epidemiological and clinical trials used OABSS as inclusion criteria, assessment of severity, and primary or secondary outcome measures^44,50,51^. The score is the simple sum of four symptom scores, which addresses daytime voiding, nighttime voiding, urgency, and urgency incontinence, with sum scores (OABSS total score) ranging from 0 to 15 points^20^. Diagnosing OAB requires urgency scores ≥ 2 and sum scores ≥ 3 on OABSS^20^. In addition, the OABSS total score is classified by a total score of 3–5 as mild, 6–11 as moderate, and 12–15 as severe^20^.

### Statistical analysis

We used version 15 of Jmp (SAS Institute Japan Inc., Tokyo) for statistical analysis. Student’s t-test for normally distributed variables, the Wilcoxon signed-rank test for non-normally distributed variables, and the chi-square test for categorical variables were used to evaluate the association between the presence (OABSS ≥ 3) or absence (OABSS < 3) of OAB. Then, using multivariable logistic analysis, we evaluated the OR of the selected variables for the presence of OAB. Finally, variables of patients with mild OAB and those with moderate to severe OAB were compared. Data are shown as means with 95% CI, unless otherwise specified. P < 0.05 was considered to be statistically significant.

## Supplementary Information

Supplementary Information.

## Data Availability

The datasets generated during and/or analyzed during the current study are not publicly available due to regulations by the Clinical Research Ethics Committee at Yotsuya Medical Cube but are available from the corresponding author on reasonable request.
