# Author Correction: Association between overactive bladder and pelvic organ mobility as evaluated by dynamic magnetic resonance imaging

**DOI:** 10.1038/s41598-021-97701-w

**Published:** 2021-09-07

**Authors:** Kurenai Kinno, Noritoshi Sekido, Yasuharu Takeuchi, Yoshitomo Sawada, Shoutarou Watanabe, Yasukuni Yoshimura

**Affiliations:** 1 Department of Urology, Toho University Graduate School of Medicine, 5-21-16 Omorinishi, Ota City, Tokyo 143-8540 Japan; 2 Department of Urology, Toho University Ohashi Medical Center, 2-22-36 Ohashi, Meguro City, Tokyo 153-8515 Japan; 3 Department of Urology, Yotsuya Medical Cube, 7-7 Nibancho, Chiyoda City, Tokyo 102-0084 Japan; 4 Female Pelvic Health Center, Showa University Northern Yokohama Hospital, 35-1 Chigasaki-chyuou, Tsuzuki Ward, Yokohama, Kanagawa 224-8503 Japan

Correction to: *Scientific Reports* 10.1038/s41598-021-93143-6, published online 02 July 2021

The original version of this Article omitted an affiliation for Kurenai Kinno. The correct affiliations for Kurenai Kinno are listed below:

Department of Urology, Toho University Graduate School of Medicine, 5-21-16 Omorinishi, Ota City, Tokyo, 143-8540, Japan

Department of Urology, Toho University Ohashi Medical Center, 2-22-36 Ohashi, Meguro City, Tokyo, 153-8515, Japan

Department of Urology, Yotsuya Medical Cube, 7-7 Nibancho, Chiyoda City, Tokyo, 102-0084, Japan

The original Article and accompanying Supplementary Information file have been corrected.

