# Supplementary material for: Association between overactive bladder and pelvic organ mobility as evaluated by dynamic magnetic resonance imaging
Source: Sci Rep. 2021 Jul 2;11:13726. doi: 10.1038/s41598-021-93143-6 (PMC8253763; doi:10.1038/s41598-021-93143-6)
Supplement: Supplementary file 1 — Supplementary Information. [file 41598_2021_93143_MOESM1_ESM.docx]

**Supplementary Information**

**Title:**

Association between overactive bladder and pelvic organ mobility as evaluated by dynamic magnetic resonance imaging

**Authors:**

Kurenai Kinno^1,2,3^, Noritoshi Sekido^2^*, Yasuharu Takeuchi^2^, Yoshitomo Sawada^2^, Shoutarou Watanabe^2^, Yasukuni Yoshimura ^3,4^

**Affiliations:**

1. Department of Urology, Toho University Graduate School of Medicine
   5-21-16 Omorinishi, Ota City, Tokyo,143-8540, Japan
2. Department of Urology, Toho University Ohashi Medical Center
   2-22-36 Ohashi, Meguro City, Tokyo 153-8515, Japan
3. Department of Urology, Yotsuya Medical Cube
   7-7 Nibancho, Chiyoda City, Tokyo 102-0084, Japan
4. Female Pelvic Health Center, Showa University Northern Yokohama Hospital
   35-1 Chigasaki-chyuou, Tsuzuki Ward, Yokohama City, Kanagawa 224-8503, Japan

**Supplementary Table S1.**

Results of multivariable logistic regression analysis for the presence or absence of overactive bladder. ARy, y coordinate of anorectal angle; BMI, body mass index; CI, confidence interval; iUSLε, strain on the imaginary line of the uterosacral ligament; OR, odds ratio; SE, standard error; χ2, chi-square test

| Model | Variables | Coefficient (B) | SE | Wald χ2 | p | OR | 95% CI |
| --- | --- | --- | --- | --- | --- | --- | --- |
| 1 |  |  |  |  |  |  |  |
|  | Intercept | -7.537 | 2.024 | 13.87 | 0.0002 |  |  |
|  | BMI | 0.125 | 0.079 | 2.53 | 0.1117 | 1.13 | 0.97, 1.32 |
|  | ARy at rest | -0.048 | 0.028 | 3.01 | 0.0826 | 0.95 | 0.90, 1.01 |
|  | iUSLε | 2.339 | 0.999 | 5.48 | 0.0192 | 10.37 | 1.46, 73.45 |
|  |  |  |  |  |  |  |  |
| 2 |  |  |  |  |  |  |  |
|  | Intercept | -7.647 | 2.397 | 10.18 | 0.0014 |  |  |
|  | Age | 0.002 | 0.024 | 0.01 | 0.9315 | 1.00 | 0.96, 1.05 |
|  | BMI | 0.125 | 0.079 | 2.54 | 0.1110 | 1.13 | 0.97, 1.32 |
|  | ARy at rest | -0.047 | 0.030 | 2.44 | 0.1183 | 0.95 | 0.90, 1.05 |
|  | iUSLε | 2.332 | 1.002 | 5.42 | 0.0199 | 10.30 | 1.45, 73.44 |

**Supplementary Table S2.**

Characteristics of patients with mild and moderate to severe overactive bladder (OAB). The p-value for numerical variables was calculated by Student’s t-test except the p-values with a superscript (*), for which the Wilcoxon rank sum test was used. BMI, body mass index; CI, confidence interval; OABSS, overactive bladder symptom score; POP-Q, pelvic organ prolapse quantification system; SCIPP, sacrococcygeal inferior pubic point

|  | Mild OAB | | Moderate to Severe OAB | |  |
| --- | --- | --- | --- | --- | --- |
|  | n=12 | | n=23 | |  |
|  | mean | 95% CI | mean | 95% CI | p |
| Age, years | 58.83 | 51.45, 66.21 | 65.17 | 60.99, 69.35 | 0.0949 |
| BMI, kg/m^2^ | 23.13 | 21.72, 24.55 | 24.82 | 23.62, 26.01 | 0.0787 |
| Parity | 2.08 | 1.45, 2.72 | 2.35 | 2.07, 2.63 | 0.6459* |
| Smoking, n | 0 |  | 0 |  |  |
| Chief complaints, n |  |  |  |  |  |
| Pelvic pressure | 3 |  | 5 |  | 1.0000 |
| Vaginal bulging | 9 |  | 18 |  |  |
| Co-morbidities, n |  |  |  |  |  |
| Diabetes | 0 |  | 2 |  | 0.5361 |
| Hypertension | 2 |  | 12 |  | 0.0697 |
| Hyperlipidemia | 2 |  | 6 |  | 0.6855 |
| POP-Q stage, n |  |  |  |  |  |
| II / III / IV | 3/9/0 |  | 5/17/1 |  | 0.7559 |
| OABSS total score | 4.08 | 3.58, 4.59 | 8.13 | 7.30, 8.96 | <0.0001* |
| SCIPP line, mm | 115.18 | 110.48, 119.88 | 114.80 | 111.15, 118.45 | 0.8951 |

**Supplementary Table S3.**

Parameters on dynamic magnetic resonance imaging in patients with mild and moderate to severe overactive bladder (OAB). x and y indicate the x and y coordinates of each pelvic organ point (AR, B, BN, and C). xx and yy indicate the distance between coordinate positions of the pelvic organ points before and during straining in x and y directions, respectively. p indicates the distance derived from the Pythagorean theorem. Note that the effect of pelvic organ mobility (POM) on the X-axis is negative when moving in the ventral direction and that of POM on the Y-axis is negative when moving in the caudal direction. Also, note that all diagonal POMs are positive. The p-value for numerical variables was calculated by Student’s t-test except the p-values with a superscript (*), for which the Wilcoxon rank sum test was used. AR, anorectal angle; AUI, angle of urethral inclination; AVWL, anterior vaginal wall length; B, most dependent position of the bladder; BN, bladder neck; C, uterine cervix; CI, confidence interval; H-line, length of the urogenital hiatus; iCL, imaginary line of the cardinal ligament; iUSL, imaginary line of the uterosacral ligament; M’-line, length of the hiatal descent; PUVA, posterior urethrovesical angle; ε, strain

|  | Mild OAB | | Moderate to Severe OAB | |  |
| --- | --- | --- | --- | --- | --- |
|  | n=12 | | n=23 | |  |
|  | mean | 95% CI | mean | 95% CI | p |
| BN, mm |  |  |  |  |  |
| At rest |  |  |  |  |  |
| BNx | 17.47 | 15.00, 19.93 | 19.74 | 17.13, 22.35 | 0.2470 |
| BNy | 4.68 | 0.22, 9.15 | 2.67 | -0.62, 5.95 | 0.4502 |
| During straining |  |  |  |  |  |
| BNx | 4.75 | -2.61, 12.11 | 2.63 | -1.62, 6.89 | 0.5737 |
| BNy | -15.93 | -22.10, -9.75 | -19.07 | -22.66, -15.47 | 0.3218* |
| Distance |  |  |  |  |  |
| BNxx | -12.72 | -20.88, -4.55 | -17.10 | -21.74, 12.47 | 0.2905 |
| BNyy | -20.61 | -25.67, -15.54 | -21.73 | -24.99, -18.48 | 0.6841 |
| BNp | 26.21 | 19.27, 33.15 | 28.78 | 24.35, 33.21 | 0.4949 |
|  |  |  |  |  |  |
| B, mm |  |  |  |  |  |
| At rest |  |  |  |  |  |
| Bx | 24.73 | 21.21, 28.26 | 30.27 | 26.95, 33.59 | 0.0342 |
| By | 10.03 | 5.01, 15.04 | 7.08 | 3.88, 10.29 | 0.2833 |
| During straining |  |  |  |  |  |
| Bx | 6.58 | -1.76, 14.93 | 5.21 | -1.56, 11.98 | 0.7973 |
| By | -33.63 | -51.51, -15.76 | -37.63 | -46.80, -28.46 | 0.6394 |
| Distance |  |  |  |  |  |
| Bxx | -18.15 | -28.09, -8.21 | -25.06 | -32.77, -17.36 | 0.2653 |
| Byy | -43.66 | -61.20, -26.12 | -44.71 | -53.37, -36.06 | 0.8976 |
| Bp | 48.80 | 30.31, 67.30 | 52.23 | 41.53, 62.94 | 0.7165 |
|  |  |  |  |  |  |
| C, mm |  |  |  |  |  |
| At rest |  |  |  |  |  |
| Cx | 45.75 | 38.60, 52.90 | 49.81 | 43.38, 56.24 | 0.4134 |
| Cy | 1.53 | -7.76, 10.82 | 2.03 | -1.16, 5.23 | 0.2373* |
| During straining |  |  |  |  |  |
| Cx | 26.93 | 14.48, 39.37 | 32.94 | 24.91, 40.97 | 0.3781 |
| Cy | -33.56 | -52.54, -14.57 | -28.33 | -35.42, -21.25 | 0.5063 |
| Distance |  |  |  |  |  |
| Cxx | -18.83 | -26.07, -11.58 | -16.87 | -22.61, -11.13 | 0.6677 |
| Cyy | -35.09 | -49.09, -21.09 | -30.37 | -36.94, -23.80 | 0.4612 |
| Cp | 41.01 | 26.67, 55.36 | 36.04 | 28.42, 43.66 | 0.4771 |
|  |  |  |  |  |  |
| AR, mm |  |  |  |  |  |
| At rest |  |  |  |  |  |
| ARx | 36.44 | 32.39, 40.49 | 37.41 | 35.06, 39.76 | 0.6409 |
| ARy | -20.89 | -27.33, -14.46 | -19.33 | -23.17, -15.48 | 0.6403 |
| During straining |  |  |  |  |  |
| ARx | 34.73 | 29.28, 40.17 | 34.00 | 30.17, 37.83 | 0.8178 |
| ARy | -39.51 | -49.08, -29.93 | -38.70 | -44.21, -33.18 | 0.8675 |
| Distance |  |  |  |  |  |
| ARxx | -1.72 | -6.65, 3.22 | -3.41 | -5.81, -1.01 | 0.4613 |
| ARyy | -18.62 | -24.72, -12.52 | -19.37 | -23.63, -15.11 | 0.8300 |
| ARp | 19.99 | 13.71, 26.28 | 20.62 | 16.56, 24.67 | 0.8555 |
|  |  |  |  |  |  |
| iUSL, mm |  |  |  |  |  |
| At rest | 69.84 | 64.72, 74.96 | 64.66 | 58.01, 71.31 | 0.2850 |
| During straining | 97.13 | 81.56, 112.70 | 88.08 | 78.99, 97.17 | 0.2614 |
| iUSL ε | 0.38 | 0.21, 0.56 | 0.38 | 0.26, 0.51 | 0.6640* |
|  |  |  |  |  |  |
| iCL, mm |  |  |  |  |  |
| At rest | 89.82 | 80.04, 99.60 | 84.29 | 78.20, 90.37 | 0.2928 |
| During straining | 128.40 | 108.82, 147.98 | 119.10 | 111.06, 127.13 | 0.2718 |
| iCL ε | 0.42 | 0.28, 0.57 | 0.43 | 0.33, 0.53 | 0.9861* |
|  |  |  |  |  |  |
| H-line, mm |  |  |  |  |  |
| At rest | 57.23 | 51.95, 62.50 | 58.26 | 55.38, 61.13 | 0.6918 |
| During straining | 62.15 | 56.84, 67.46 | 63.77 | 59.66, 67.89 | 0.6640* |
|  |  |  |  |  |  |
| M'-line, mm |  |  |  |  |  |
| At rest | 29.53 | 24.58, 34.49 | 29.78 | 27.28, 32.27 | 0.9166 |
| During straining | 38.58 | 34.20, 42.97 | 41.77 | 37.02, 46.52 | 0.3684 |
|  |  |  |  |  |  |
| AVWL, mm |  |  |  |  |  |
| At rest | 39.02 | 30.25, 47.78 | 37.49 | 31.54, 43.44 | 0.7577 |
| During straining | 70.33 | 52.22, 88.45 | 77.94 | 62.87, 93.01 | 0.3135* |
| Change | 31.32 | 12.77, 49.86 | 40.45 | 26.97, 53.92 | 0.4068 |
|  |  |  |  |  |  |
| PUVA, degree |  |  |  |  |  |
| At rest | 136.40 | 118.69, 154.11 | 132.79 | 121.18, 144.39 | 0.7114 |
| During straining | 121.82 | 95.82, 147.82 | 110.19 | 93.80, 126.57 | 0.4075 |
| Change | -14.58 | -48.77, 19.60 | -22.60 | -43.30, -1.90 | 0.3053* |
|  |  |  |  |  |  |
| AUI, degree |  |  |  |  |  |
| At rest | 24.08 | 18.98, 29.19 | 25.83 | 21.13, 30.52 | 0.8890* |
| During straining | 112.50 | 103.18, 121.82 | 104.65 | 100.44, 108.86 | 0.1175* |
| Change | 88.42 | 81.42, 95.41 | 78.83 | 72.38, 85.27 | 0.0582 |
